# Papillary Tumor of the Pineal Gland: Series of Four Clinical Cases

**DOI:** 10.7759/cureus.61308

**Published:** 2024-05-29

**Authors:** Martha Lilia L Tena Suck, Jose Gabriel Rojo Alfaro, Erick Gomez Apo, Daniel Rembao Bojórquez, Eliezer Villanueva-Castro, José Alfredo Castro Ibañez

**Affiliations:** 1 Department of Neuropathology, Instituto Nacional de Neurología y Neurocirugía Manuel Velasco Suárez, Mexico City, MEX; 2 Department of Neurophysiology, Instituto Nacional de Neurología y Neurocirugía Manuel Velasco Suárez, Mexico City, MEX; 3 Department of Pathology, Hospital General de Mexico, Mexico City, MEX; 4 Department of Neuropathology, Instituto Nacional de Neurología y Neurocirugía Manuel Velasco Suárez, Mexico city, MEX; 5 Department of Neurosurgery, Instituto Nacional de Neurología y Neurocirugía Manuel Velasco Suárez, Mexico City, MEX; 6 Departamento de Neuroimagen, Instituto Nacional de Neurología y Neurocirugía Manuel Velasco Suárez, Mexico City, MEX

**Keywords:** immunohistochemistry, brain papillary tumors, primary tumors of the pineal region (tprs), central nervous system, papillary tumor of the pineal region

## Abstract

The papillary tumor of the pineal region (PTPR) is a rare neuroepithelial tumor originating from specialized ependymocytes. It primarily affects structures within the pineal region, including the pineal gland, epithalamus, quadrigeminal cistern, and posterior wall of the third ventricle. Here, we present a series of four cases characterized by symptoms associated with obstructive hydrocephalus such as headaches, seizures, visual disturbances, gait disturbances, and Parinaud syndrome. Imaging studies revealed lesions in the pineal region, prompting surgical intervention. Histopathological examination, including biopsy and intraoperative analysis, confirmed the diagnosis of PTPR. Despite advancements, the etiology and pathogenesis of PTPR remain incompletely understood, warranting further research to refine management strategies.

## Introduction

The term 'pineal region' commonly refers to an area encompassing the pineal gland and surrounding structures such as the epithalamus, quadrigeminal cistern, and the posterior wall of the third ventricle. This region holds significance due to the presence of both malignant and benign tumors, attributed to the diverse array of cells and tissues expressed in this area. Primary tumors of the pineal region constitute a histologically heterogeneous group, including neoplasms of the pineal parenchyma, germ cell neoplasms, tumors arising from adjacent structures, and pineal metastases [1-4].

The epidemiology of papillary tumors of the pineal gland is particularly intriguing. Tumors of the pineal region constitute approximately 0.4-1% of primary central nervous system (CNS) tumors. Within this spectrum, pineal region tumors can manifest across various age groups, including children, where they represent 3% to 8% of intracranial neoplasms, as well as in young and middle-aged adults [5]. While most tumors in the pineal region show a slight predilection for women over men, papillary tumors of the pineal region (PTPR) exhibit no significant gender predilection. The onset of PTPR can occur across a wide age range, spanning from 5 to 66 years. Notably, PTPR has a propensity to metastasize to other parts of the central nervous system. However, there remains a lack of comprehensive information on its clinical behavior, highlighting the necessity for further studies in this area [3].

The first description of PTPR was conducted by Jouvet et al. in 2003 [6]. Since then, approximately more than 100 cases of PTPR have been reported to date [7,8]. The fourth edition of the World Health Organization's (WHO) classification of central nervous system tumors, published in 2007, introduced several new entities, including the papillary tumor of the pineal region. This tumor was categorized as grade II or III due to its likely malignancy and aggressive clinical behavior [2].

According to the new WHO classification in 2021, tumors of the pineal parenchyma encompass five distinct types: pineocytoma (PC), categorized as grade I; pineal parenchymal tumors of intermediate differentiation (PPTID), ranging from grade I to II; papillary tumor of the pineal region (PTPR), categorized as grade II to III; pineoblastoma (PB), recognized as the most aggressive high-grade tumor or grade III; and desmoplastic/mixed pineal region tumor, classified as grade I. Additionally, germ cell tumors are also included within this classification [9]. The 2021 WHO classification introduced a new entity: desmoplastic/mixed tumor of the pineal region. This classification is based on the presence of the SMARCB1 mutant mutation and the absence of histological markers of malignancy [9].

The clinical presentation of papillary tumors of the pineal region varies depending on size and location. The most common symptoms are associated with obstructive hydrocephalus and include headaches (79%), visual disturbances (61%), and gait disturbances (27%) [3,10]. Other symptoms may include diplopia, vomiting, seizures, lethargy, and neurological signs, such as Argyll Robertson pupils and Parinaud syndrome, attributed to compression of the dorsal midbrain [10,11].

## Case presentation

Case 1

**Figure 1:**
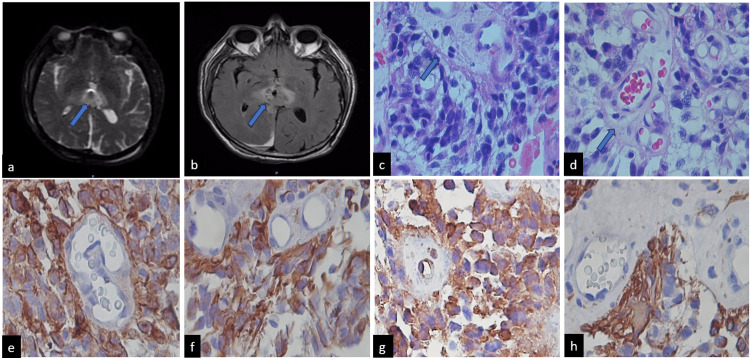
Characteristics of Case 1 Magnetic resonance imaging (MRI) in T2 (a). MRI in FLAIR (b). Histologically, the tumor exhibits papillary structures with hypovascularized fibroconnective nuclei, and vessels with typical characteristics (c) and (d). Epithelial-like cells with hyperchromatic nuclei displaying cellular atypia are also observed (hematoxylin and eosin x400). Blue arrows indicate a vascularized fibroconnective core. Immunohistochemistry revealed positivity for EA1/EA3 (e), synaptophysin (f), Nestin (g), and GFAP (h), highlighting densely fibrillar areas (immunohistochemistry x400).

In September 2021, a 61-year-old male presented with memory issues, apraxia of gait, sphincter dysfunction, and weakness on the right side of the body. Imaging studies revealed a pineal lesion and hydrocephalus (Figure 1).

Subsequently, the patient underwent ventriculoperitoneal shunt system (VPS) placement, resulting in an improvement in symptoms. However, in January 2022, the patient experienced a generalized tonic-clonic seizure lasting approximately two minutes, prompting a referral to our institution.

Upon physical examination, the patient was postictal, exhibiting drowsiness, eye-opening in response to verbal stimuli, incomprehensible speech, and partial responsiveness to simple commands.

A follow-up CT scan showed an enlargement of the lesion and increased ventricular dilation, despite apparently normal valve function, compared to previous imaging studies.

A diagnosis of obstructive hydrocephalus secondary to a lesion in the third ventricle, possibly a germinoma, was established. This led to further evaluation, including endoscopic examination, review of the shunt system, septostomy, and biopsy, The tumor measured 35x20 mm. 

The histopathological analysis identified the lesion as a papillary pineal tumor, classified as Grade II according to WHO standards. Immunohistochemistry staining revealed positive results for S100, synaptophysin, Nestin, CD99, GFAP, vimentin, CD117, cytokeratin 18, and EA1/EA3. Additionally, increased expression of neurofilaments and synaptophysin was observed while GFAP, chromogranin, and CD34 staining in vessels was negative (Figure 1).

Subsequently, a radiosurgery protocol was initiated, receiving 22 sessions and resulting in improved speech, movement, and comprehension, as well as resolution of recurrent seizures and enhancement in sleep quality. Four years later, the patient is still alive.

Case 2

**Figure 2:**
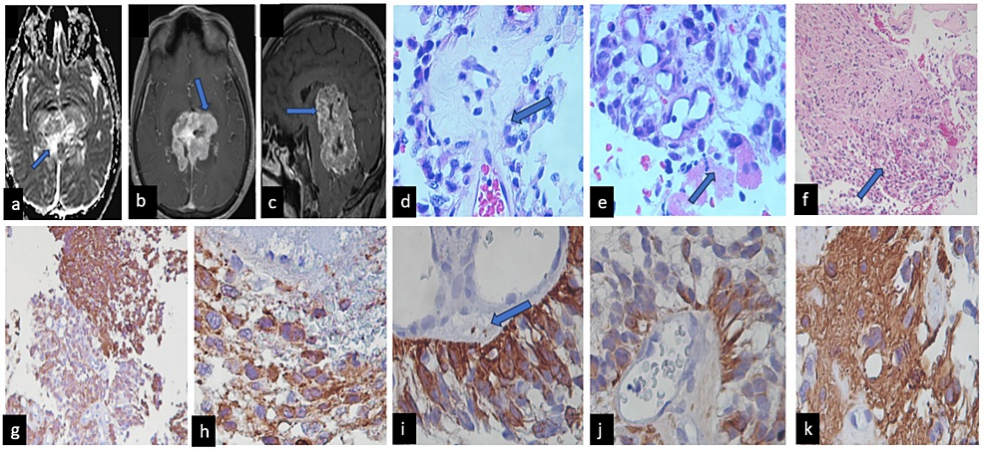
Characteristics of Case 2 MRI showed (a) extra-axial images located in the pineal region and extending toward the posterior fossa. It is irregular, with lobed, defined margins, heterogeneous signal intensity, and chemical/necrotic degeneration areas inside. It is predominantly hypointense. On T2 and FLAIR (2b and 2c), it presents a slight increase in signal intensity and multiple central and peripheral flow voids. (d) Histologically tumor formed by epithelial-like cells that form papillae is observed (H&E x 400), the central fibrovascular core showed varied hyalinization, and in (e) observed vessels proliferations and some cells with cellular atypia (H&Ex400). (f) Necrosis was observed (H&E x 200). Immunohistochemistry showed (g) synaptophysin-positive cells, (h), Nestin-positive cells, (i) CD117 showed focally positive cells, (j) AE/1AE3 was also positive, and (k) GFAP was fibrillary stromal positivity immunoexpression (IHQ x400 original magnifications).

In September 2020, a 46-year-old male experienced a severe generalized headache, for which he received unspecified pharmacological management. By November, he began experiencing gait alterations and disorientation. By December, he developed bilateral hearing and vision loss. Subsequent investigations revealed a pineal tumor accompanied by hydrocephalus. In March 2021, he underwent placement of a ventriculoperitoneal (VP) shunt and tumor resection (Figure 2).

The patient's balance improved post-resection, allowing him to carry out daily activities. However, eight months later, he presented with lower limb weakness, requiring support for walking, along with hypoxemia, loss of sphincter control, hypersomnia, medication refusal, and a lack of communication intent. He experienced tonic-clonic seizures.

During the physical examination, the patient appeared awake but demonstrated a lack of directed attention, fixation, or sustained focus. The assessment of the vestibulocochlear nerve (VIII) was inconclusive. In the motor examination, increased tone and normal tropism were observed. Gait assessment was initially not feasible.

Over the subsequent days, the patient's condition evolved. He presented an ocular opening response to nociceptive stimuli, demonstrating a localized pain response in both thoracic limbs. Additionally, the patient produced incomprehensible sounds.

The MRI showed extra-axial images are observed, located in the pineal region and extending toward the posterior fossa. It is irregular, with lobed, defined margins, heterogeneous signal intensity, and chemical/necrotic degeneration areas inside. On MRI (2a, 2b, 2c), it is predominantly hypointense. On T2 (2a) and FLAIR (2b and 2c), it presents a slight increase in signal intensity and multiple central and peripheral flow voids. In SWI, there are some central irregular areas of signal deviation; It does not restrict diffusion after contrast administration (T1 with contrast) and it presents intense and heterogeneous enhancement. This injury causes compression of the ventricular system; it compresses and displaces the thalami and lenticular nuclei toward the rostral and dorsal direction, the body and splenium of the corpus callosum toward the dorsal direction, the brain stem in the ventral direction and the cerebellum toward the caudal direction, causing edema of the adjacent parenchyma and herniation of the cerebellar tonsils. Its dimensions are 72x45x60 mm in the dorsoventral, laterolateral, and rostro caudal axes.

Histopathological analysis characterized the lesion as a pineal gland lesion with a papillary pineal tumor featuring a solid component graded II according to WHO standards. Immunohistochemistry staining revealed positive results for GFAP, cytokeratin, EMA, SNE, synaptophysin, Nestin, CD99, vimentin, CD117, CK/18 EA1/EA3, chromogranin in neoplastic cells, and CD34 in vessels, while neoplastic cells were negative. Neurofilaments and synaptophysin showed increased expression.

A follow-up CT scan revealed the presence of a residual tumor and a shunt valve, demonstrating hyperfunctionality with ventricular collapse. Unfortunately, despite therapeutic interventions, surgery for tumor residue removal was deemed unfeasible due to a low postoperative functional prognosis. The patient deteriorated neurologically and passed away 6 months later, having not received radiotherapy.

Case 3

**Figure 3:**
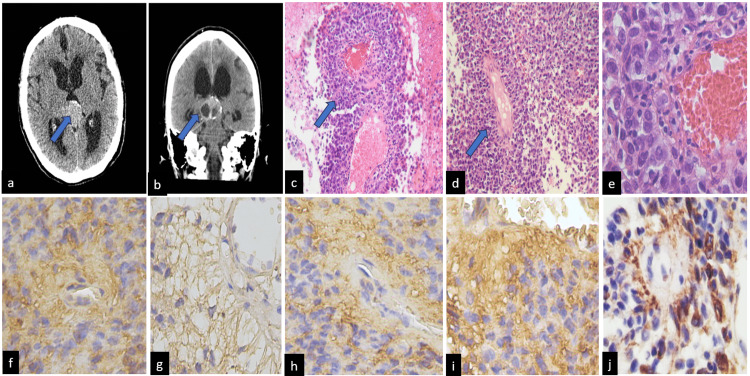
Characteristics of Case 3 The cerebral CT scan showed a pineal tumor with a cyst appearance in (a) and (b). Histologically, the tumor was formed by epithelial-like cells covering a papillary structure with a vascularized fibroconnective core in (c) and (d), (H&Ex200), and (e) and observed the morphological characteristics of the epithelial-like cells (H&E x400). The blue arrows showed the fibroconnective tissue core. The immunohistochemistry showed positivity immunoexpression for AE/1AE3 in (f), the GFAP was fibrillary appearance (g), CK/18 was weak with a fibroconnective vascularized core predominance in (h), synaptophysin positive cells in (i), and CD117 was focally positive cells in (j) (IHQ x400).

A 58-year-old female presented to our clinic with a one-month history of progressive headache, visual disturbance, and gait impairment. Neurological examination revealed marked ataxia but no further neurological deficits were observed. MRI of the brain showed obstructive hydrocephalus caused by a tumor located at the third ventricle, affecting the pineal region (Figure 3).

The patient underwent surgical resection of the tumor. the tumor measured 34x25 mm with a cystic aspect. Histologically, the tumor comprised small blue cells, ranging from round to slightly medium-sized, with evident mitotic figures and cellular atypia. These cells demonstrated a perivascular distribution and formed pseudo-papillary structures, creating a pseudo-rosette appearance reminiscent of natural stems and papillary fibroblasts. While some areas displayed features resembling ependymoma, immunohistochemistry revealed positive staining for SNE, synaptophysin, Nestin, CD99, GFAP, vimentin, CD117, CK/18, and EA1/EA3 (Figure 3). 

Based on histological images and an immunohistochemistry panel, it was diagnosed as a papillary tumor of the pineal gland, The patient received incomplete radiotherapy and discontinued treatment and follow-up by personal choice.

Case 4

**Figure 4:**
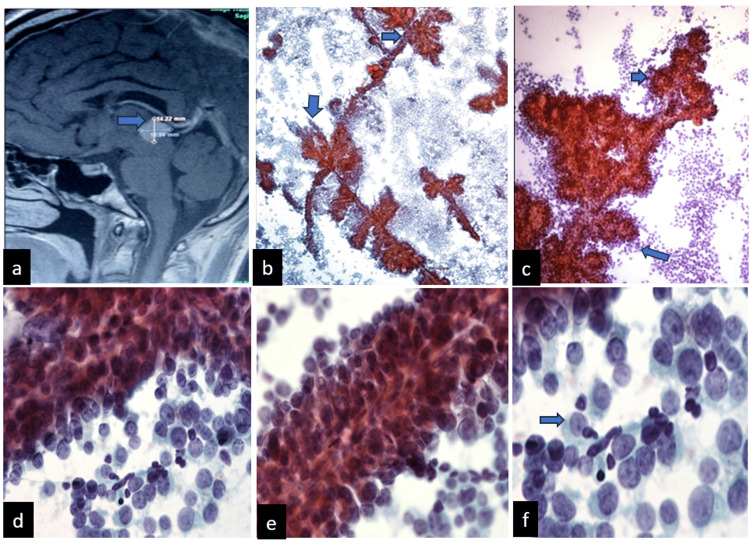
Characteristics of Case 4 (a) The sagittal section of MRI in the T1 sequence shows a well-defined, hyperintense lesion in the pineal region. (b) Cytological scrunch smear, panoramic photograph showing moderate cellularity, with papillary structures (Pap smear, 400X). (c) These papillae are a bit complex in some areas, with branching of the papillae (400X). Cytological details and the stratification of neoplastic cells and scattered cells are identified. The scattered cells are medium, with granular chromatin, inconspicuous nucleoli, and slight nuclear irregularity; the cytoplasm is mild to moderate (d), (e), and (f) (Papanicolaou stain 100X).

A 20-year-old man presented with a clinical history spanning a few months, characterized by headache, progressive obstructive hydrocephalus, and Parinaud syndrome. He was admitted to our hospital for a stereotaxic biopsy protocol. Nuclear magnetic resonance imaging revealed a lesion measuring 14.94 x 14.22 centimeters in the pineal region, characterized by hyperintensity, uniformity, an oval shape, and well-defined borders. Cytological examination revealed a moderately cellular cytology, consisting of papillae lined by medium-sized cells with scant cytoplasm, nuclear contours showing slight irregularity, and granular chromatin (Figure 4).

The patient discontinued treatment and follow-up by personal choice, a definitive specimen was not sent to the neuropathology service, and immunohistochemistry stains were not performed.

## Discussion

Although the name of papillary tumor of the pineal region (PTPR) suggests the involvement of cells within the pineal gland, such as pinealocytes, phagocyte cells, peptidergic neurons, and interstitial cells, current understanding indicates that PTPR originates from ependymocytes. It is noteworthy that the expression of proteins in PTPR is presumed to be ependymal [12,13].

Imaging studies frequently encounter challenges in determining the origin of tumors within the complex pineal region. The intricate structures present in this area often obscure the specific type of tumor, underscoring the importance of employing comprehensive diagnostic approaches. A nuanced understanding of the complexities associated with the pineal region is crucial for achieving accurate diagnosis and developing tailored treatment plans.

In the initial case, the evolution of the patient's symptoms and their correlation with MRI findings initially suggested a diagnosis of germinoma. However, as demonstrated in these cases, the clinical manifestations of PTPR are nonspecific, complicating a definitive diagnosis. The difficulty lies in differentiating PTPR from other tumors of pineal parenchymal origin, such as pineocytoma (PC), pineoblastoma (PB), and germ cell tumors, particularly germinomas. The differential diagnosis of PTPR must also consider other pineal region tumors, including teratomas, glial tumors, vascular malformations, and aneurysms [1].

PTPR macroscopically presents as relatively large (2.5-4 cm), well-circumscribed tumors, which are grossly indistinguishable from pineocytomas and other pineal region tumors. MRI imaging often reveals a well-circumscribed tumor, frequently accompanied by cystic components and hyperintensity on T1-weighted images, which is the most characteristic imaging feature [14,15]. PTPRs typically manifest as circumscribed lesions with variable T1 and T2 signals, often exhibiting post-contrast enhancement. Given the potential for cerebrospinal fluid (CSF) dissemination, it is advisable to perform a contrast-enhanced MRI of the entire craniospinal axis. This comprehensive imaging approach is crucial for detecting local tumor recurrence and assessing for dissemination to the leptomeninges [15].

Histologically, papillary tumors of the pineal region are characterized by epithelial growth patterns. However, the accurate diagnosis of this neoplasm can be challenging due to its resemblance to other primary or secondary papillary lesions within the pineal region. These include parenchymal pineal tumors, papillary ependymoma, papillary meningioma, choroid plexus papilloma, and metastatic papillary carcinoma [1].

PTPRs are composed of cells that are typically large, columnar, and frequently exhibit prominent nuclei. These cells form papillary structures, rosettes, and pseudo-rosettes, often enveloping vessels with a layer of these cells [6,7]. Elevated mitotic and proliferative activity is correlated with a greater risk of recurrence and poorer outcomes in patients with PTPR [3]. In three cases, a papillary morphology was observed, while one exhibited a distinctive cytological appearance characterized by a papillary image with evident cellular atypia. Although a definitive tissue study was not feasible, the cytological findings were highly suggestive of papillary tumor morphology. Smears exhibited hypercellularity with numerous papillary tissue fragments and single cells, predominantly displaying epithelial-like morphology with varying degrees of papillary formation and cellular pleomorphism. Notably, these cells exhibited a plasmacytoid appearance and cytoplasmic fragility [16]. Numerous cells exhibit foamy, eosinophilic, or clear, occasionally vacuolated cytoplasm. Mitotic figures are rare, and areas of necrosis have been observed. Papillary fragments are characterized by an evident inner or central vascular core. Neoplastic cells display moderate pleomorphism, and the background exhibits a foamy, lace-like, 'tigroid' appearance. This 'tigroid' appearance, observed in germinoma and related tumors, is a characteristic cytologic feature. While it can also be seen in other clear-cell, glycogen-rich tumors, PTPR shares its clear-cell morphology and PAS-positive cytoplasmic granules with these neoplasms [16].

Immunohistochemically, positive staining is commonly observed for cytokeratin, Nestin, S-100 protein, neuron-specific enolase (NSE), and vimentin in PTPR. However, staining for epithelial membrane antigen (EMA) and glial fibrillary acidic protein (GFAP) is typically weak or negative [6,7,16]. The Ki-67 labeling index was found to be relatively low in all cases. Based on histological and immunohistochemical findings, a primary consideration for the differential diagnosis should be a papillary tumor of the pineal region (PTPR) [14,15]. Tumors forming papillary structures, such as choroid plexus tumors (CPT), typically exhibit positive immunoreactivity for CK+, GFAP+, transthyretin (TTR)+, and KIR7.1+. On the other hand, ependymomas demonstrate immunoreactivity for AE1/AE3 keratin, GFAP, S100, CD99, and vimentin, and may exhibit ZFTA-RELA or YAP1 fusions. Additionally, they may display mixed mucoid material highlighted by PAS and positive Alcian blue staining. These tumors are typically negative for synaptophysin, neurofilaments, NSE, CK, and desmin [9].

In our report of four cases, comprising three males and one female, we observed that the natural course of the disease is uncertain. Three patients received radiotherapy; one showed improvement and adequate recovery, two discontinued treatment and follow-up by personal choice, and one faced functional decline despite treatment, which exhibited greater cellular atypia and necrosis, and died within six months. Recently, discussions on essential prognostic features have emerged, including the controversial possibility that the placement of an internal ventricular shunt could increase the risk of metastasis [1].

On the other hand, Fèvre-Montange et al.'s study revealed a heightened risk of symptomatic relapse in patients with incomplete resection of PTPR. While these observations underscore the importance of optimizing treatment methods, further research is warranted to fully understand their implications. The second reported case described a symptomatic relapse in a patient previously subjected to tumor resection, with subsequent studies revealing residual tumor tissue. This case aligns with findings from previous studies, emphasizing the complexities in managing PTPR. The lack of established optimal therapy for PTPR contributes to variations in treatment approaches. Individualized neurosurgical and oncological evaluations, consideration of histological patterns, immunohistochemistry, and other factors all influence treatment decisions, highlighting the need for comprehensive and tailored management strategies.

In most cases, surgery, whether total or partial tumor resection, is the primary treatment choice for PTPR, as it has been shown to improve survival rates. However, despite surgical intervention, there is a high risk of recurrence, with rates reported at 70% at six years and 58% at five years. Therefore, radiotherapy plays a crucial role in treatment, although as monotherapy, it does not significantly impact survival outcomes. However, when integrated into multidisciplinary treatment approaches, radiotherapy can help prevent tumor regression. Interestingly, in cases where surgery and radiotherapy are ineffective, chemotherapy has demonstrated success in achieving long-lasting tumor regression. The final diagnosis of PTPR relies on both predominant papillary morphology and immunohistochemical results. While PTPR should be considered in the differential diagnosis of pineal tumors, its natural history, therapeutic strategies, and prognosis remain subjects of controversy.

## Conclusions

PTPR represents a rare, yet impactful, entity among brain tumors. Despite the less clear epidemiology compared to other pineal tumors, PTPR exerts a considerable toll on patient health. These intriguing cases highlight the intricate diagnostic process involved in identifying this rare tumor, which necessitates comprehensive imaging studies and histopathological evaluation. The diagnosis and management of PTPR pose significant challenges, as it can mimic a diverse array of tumors with similar clinical and radiological features. Regrettably, the lack of established diagnostic techniques and timely interventions complicates prognostication for patients and their families. While PTPR remains a rare occurrence, its significance should not be underestimated. Early and accurate diagnosis, coupled with appropriate treatment, can markedly improve patient outcomes. Therefore, concerted efforts are needed to enhance understanding and awareness of PTPR among healthcare professionals and the broader medical community.
